# Less improvement following meniscal repair compared with arthroscopic partial meniscectomy: a prospective cohort study of patient-reported outcomes in 150 young adults at 1- and 5-years’ follow-up

**DOI:** 10.1080/17453674.2021.1917826

**Published:** 2021-04-30

**Authors:** Kenneth Pihl, Martin Englund, Robin Christensen, L Stefan Lohmander, Uffe Jørgensen, Bjarke Viberg, Jakob Vium Fristed, Jonas B Thorlund

**Affiliations:** a Department of Sports Science and Clinical Biomechanics, University of Southern Denmark, Odense, Denmark;; b Lund University, Faculty of Medicine, Department of Clinical Sciences Lund, Orthopedics, Clinical Epidemiology Unit, Lund, Sweden;; c Musculoskeletal Statistics Unit, the Parker Institute, Bispebjerg and Frederiksberg Hospital, Denmark;; d Research Unit of Rheumatology, Department of Clinical Research, University of Southern Denmark, Odense University Hospital, Odense, Denmark;; e Lund University, Faculty of Medicine, Department of Clinical Sciences Lund, Orthopedics, Lund, Sweden;; f Department of Orthopedics and Traumatology, Odense University Hospital, Odense, Denmark;; g Department of Orthopedics, Lillebaelt Hospital, Kolding, Denmark;; h Department of Orthopedics, Lillebaelt Hospital, Vejle, Denmark;; i Research Unit for General Practice, Department of Public Health, University of Southern Denmark, Odense, Denmark

## Abstract

Background and purpose — Meniscal repair may reduce long-term risk of knee osteoarthritis compared with arthroscopic partial meniscectomy (APM), whereas patient-reported outcomes may be poorer at short term than for APM. We compared patient-reported outcomes in young adults undergoing meniscal repair or APM up to ∼5 years after surgery.

Patients and methods — We included 150 patients aged 18–40 years from the Knee Arthroscopy Cohort Southern Denmark (KACS) undergoing meniscal repair or APM. Between-group differences in change in a composite of 4 of 5 Knee injury and Osteoarthritis Outcome Score (KOOS) subscales (pain, symptoms, sport and recreation, and quality of life—KOOS_4_) from baseline, 12, and 52 weeks, and a median of 5 years (range 4–6 years) were analyzed using adjusted mixed linear models, with 52 weeks being the primary endpoint.

Results — 32 patients had meniscal repair (mean age 26 [SD 6]), and 118 patients underwent APM (mean age 32 [SD 7]). The repair and APM groups improved in KOOS_4_ from before to 52 weeks after surgery (least square means 7 and 19, respectively; adjusted mean difference –12, [95% CI –19 to –4] in favor of APM). Both groups improved further from 52 weeks to 5 years after surgery with the difference in KOOS_4_ scores between the groups remaining similar.

Interpretation — Patients having meniscal repair experienced less improvements in patient-reported outcomes from baseline to 52 weeks and 5 years post-surgery. The findings highlight the need for randomized trials comparing these interventions in terms of patient-reported outcomes and knee OA development.

Recent studies have reported that arthroscopic partial meniscectomy (APM) is associated with increased risk of osteoarthritis (OA) development and knee replacement surgery as compared with knees with meniscal tears left in situ (Roemer et al. 2017, Rongen et al. 2017). Consequently, meniscal repair, which aims to preserve the meniscal tissue and thereby reduce knee OA risk, has been strongly advocated in recent years, especially for younger individuals with traumatic meniscal tears (Kopf et al. 2020). However, meniscal repair often requires longer rehabilitation time, and has a higher reoperation rate compared with APM, suggesting a trade-off between the 2 procedures (Paxton et al. 2011, Cavanaugh and Killian 2012).

Currently, the evidence of the protective ability of meniscal repair against OA compared with APM is limited to retrospective observational data (Stein et al. 2010, Lutz et al. 2015, Persson et al. 2018). Similarly, reliable information on differences in patient-reported outcomes between meniscal repair and APM is scarce, and results from the few retrospective studies are conflicting and lack assessment of change over time (Stein et al. 2010, Paxton et al. 2011, Lutz et al. 2015).

The number of meniscal repairs is increasing in accordance with current guidelines (Kopf et al. 2020). While awaiting a randomized trial evaluating knee OA development and patient-reported outcomes following meniscal repair compared with APM, we used a prospective study design with pre-specified outcomes to compare change in patient-reported outcomes in patients aged 18–40 years undergoing meniscal repair or APM at multiple time points up to between 4 and 6 years after surgery.

## Patients and methods

This prospective cohort study is described in a published protocol (Thorlund et al. 2013) and registered at ClinicalTrials.gov (NCT01871272). We followed the STROBE guideline for reporting the study.

### Patient selection

We included patients from the Knee Arthroscopy Cohort Southern Denmark (KACS) (Thorlund et al. 2013). Patients in KACS were consecutively recruited at 4 public hospitals in the region of Southern Denmark between February 1st, 2013 and January 31st, 2014, and at 1 of the initial 4 hospitals in the period February 1, 2014 to January 31, 2015. To be included in this study, patients needed to be 18–40 years of age, assigned for knee arthroscopy on suspicion of a meniscal tear by an orthopedic surgeon (i.e., based on history of injury, clinical examination, and magnetic resonance imaging [MRI] if considered necessary), able to read and understand Danish, and having an e-mail address. Patients were excluded if not having a meniscal tear at surgery, had previous or planned anterior or posterior cruciate ligament (ACL or PCL) reconstruction surgery in either knee, fracture(s) in lower extremities within 6 months before recruitment, or were unable to reply to an online questionnaire due to mental impairments.

### Patient-reported outcomes

Participant characteristics and symptom information was collected using online questionnaires before surgery (median 6 days, IQR 2–9 days) and at 12 and 52 weeks, and median 5 years (range 4–6 years) after surgery.

The main outcome was a composite score of 4 of the 5 subscales from the Knee injury and Osteoarthritis Outcome Score (KOOS), defined as KOOS_4_. The 4 subscales were: pain, symptoms, sport and recreation function (Sport/Rec), and knee-related quality of life (QOL) excluding the activities of daily living (ADL) subscale, because of ceiling effects in younger active populations (Collins et al. 2016). The KOOS is a knee-specific patient-reported outcome and each subscale ranges from 0 to 100, with 0 representing extreme knee problems and 100 representing no knee problems (Roos et al. 1998). It has been validated in individuals with traumatic knee injuries, including individuals undergoing arthroscopic meniscal surgery (Roos et al. 1998), and KOOS_4_ has been used in a previous trial assessing the effect of APM surgery (Kise et al. 2016). The main outcome was at 52 weeks (Thorlund et al. 2013), while KOOS_4_ scores 5 years after surgery and all 5 KOOS subscales were included as additional outcomes.

Other additional outcomes were Patient Acceptable Symptom State (PASS), treatment failure, knee problems after surgery, and subsequent surgery. PASS was assessed with the question: “When you think of your knee function, would you consider your current condition as satisfying? By knee function, you should take into account your activities of daily living, sport and recreational activities, your pain and other symptoms and your quality of life” with response options “yes” or “no” (Ingelsrud et al. 2015). Patients unsatisfied with their current knee function after surgery were then asked a second question relating to treatment failure: “Would you consider your current state as being so unsatisfactory that you consider the treatment to have failed?” with the response options “yes” or “no.” Subsequent surgery on the index knee was assessed using 2 questions in combination: “Have you had problems with your knee after the operation?” and “Have you had additional knee surgery because of your knee problems?” both with the response options “yes” or “no.” The latter question was only asked of those replying “yes” to having had knee problems.

### Surgical information

Surgical information was recorded at arthroscopy. A modified version of the International Society of Arthroscopy, Knee Surgery and Orthopaedic Sports Medicine (ISAKOS) classification of meniscal tears (Anderson et al. 2011) was used for the description of the surgical procedure (i.e., repair and/or APM), classification of meniscal pathology (i.e., tear type, tear location, etc.), while the International Cartilage Repair Society (ICRS) grading system (Brittberg and Winalski 2003) was used for classification of compartment-specific cartilage damage (ranging from 0 [normal cartilage] to 4 [very severe cartilage damage]).

### Statistics

As reported in the study protocol, a sample size of 67 in the APM group and 33 in the repair group would yield a power of 0.88 to detect a difference between groups of 10 points in KOOS_4_, assuming a common standard deviation of 15 and a significance level of 0.05 (Thorlund et al. 2013). Under the same assumptions the actual sample of 150 patients (118 having APM and 32 having repair) yielded a power of 0.91 to detect a 10-point difference. To reach a sufficient number of patients with repair, the original recruitment period was extended from 1 to 2 years.

For the main outcome, the difference between groups in KOOS_4_ change from baseline to 52 weeks was analyzed using a mixed linear model (REstricted Maximum Likelihood estimation [REML]) with patients as random effects and group (repair vs. APM) and time (pre-surgery, 12 weeks, 52 weeks, and 5 years), and the interaction between group and time as fixed effects. The main model was adjusted for the following pre-surgery covariates: age, sex, BMI, and preoperative KOOS_4_ score. The same analysis approach was repeated for all additional KOOS subscales separately. The underlying assumptions for the mixed linear models were assessed using residual plots and kernel density plots. Results are reported as least squares means, and differences between these with 95% confidence intervals (CI).

Additional sensitivity analyses included: (1) repeating the main analyses including only patients with traumatic meniscal tears as originally protocolized (Thorlund et al. 2013) ; (2) repeating the main analyses excluding patients having both repair and APM performed; (3) repeating the main analyses excluding patients with partial or total ACL rupture; (4) repeating the main analyses excluding patients who had had subsequent surgery on the index knee during the 5-year follow-up; and (5) repeating the main analyses adjusted for covariates with a potential difference in distributions between groups larger than 0.50 SD units (based on standardized mean differences) (Imbens and Rubin 2015). We applied the following pragmatic definition of what makes a confounding variable (C), it is likely an ancestor (cause) of the outcome (Y); it probably causes the exposure (i.e., group). Finally, in order to prevent us from adjusting for pre-existing differences (i.e., Lord’s paradox) or introducing collider bias, a potential deconfounding covariate (C) cannot be a descendant (i.e., an effect) of the exposure (group) or outcome (e.g., KOOS_4_) (Greenland 2003).

We also conducted a subgroup analysis repeating the main analyses in which patients considered ineligible for repair were excluded (i.e., patients with tears not being non-degenerative longitudinal-vertical tears located in the red–red or red–white zone). As for the main analyses, this subgroup analysis was repeated excluding patients who had had subsequent surgery on the index knee.

Lastly, in patients with complete data, the difference in proportions of patients who were unsatisfied after surgery (i.e., PASS), indicating treatment failure, or subsequent surgery between those having repair or APM, was tested by the calculation of risk ratios and risk differences with CI.

### Ethics, funding, and potential conflicts of interest

Written informed consent to participate in KACS was obtained from all patients, while the Regional Scientific Ethics Committee waived the need for ethical approval after reviewing the outline of KACS (Thorlund et al. 2013).

This study was supported by an individual postdoctoral grant (JBT) from the Danish Council for Independent Research/Medical Sciences and funds from the Region of Southern Denmark. RC acknowledge that the Parker Institute, Bispebjerg and Frederiksberg Hospital is supported by a core grant from the Oak Foundation (OCAY-18-774-OFIL).

BV reports personal fees from Osmedic Swemac and Zimmer Biomet outside the submitted work. JBT reports grants from Pfizer outside the submitted work. ME reports personal fees from Pfizer outside the submitted work. RC reports honorarium to employer from (1) Lectures: Research Methods (Pfizer, DK; 2017); GRADE Lecture (Celgene, DK; 2017), Ad Board Lecture: CAM (Orkla Health, DK; 2017); Diet in RMD (Novartis, DK; 2019); Ad Board Lecture: GRADE (Lilly, DK; 2017); Network MAs (LEO; 2020) and ((Mundipharma, 2019) and (3) Consultancy Reports: Network MAs (Biogen, DK; 2017), other from); GRADE (Celgene, 2018) all outside the submitted work. The remaining authors have nothing to declare.

## Results

**Figure 1.**
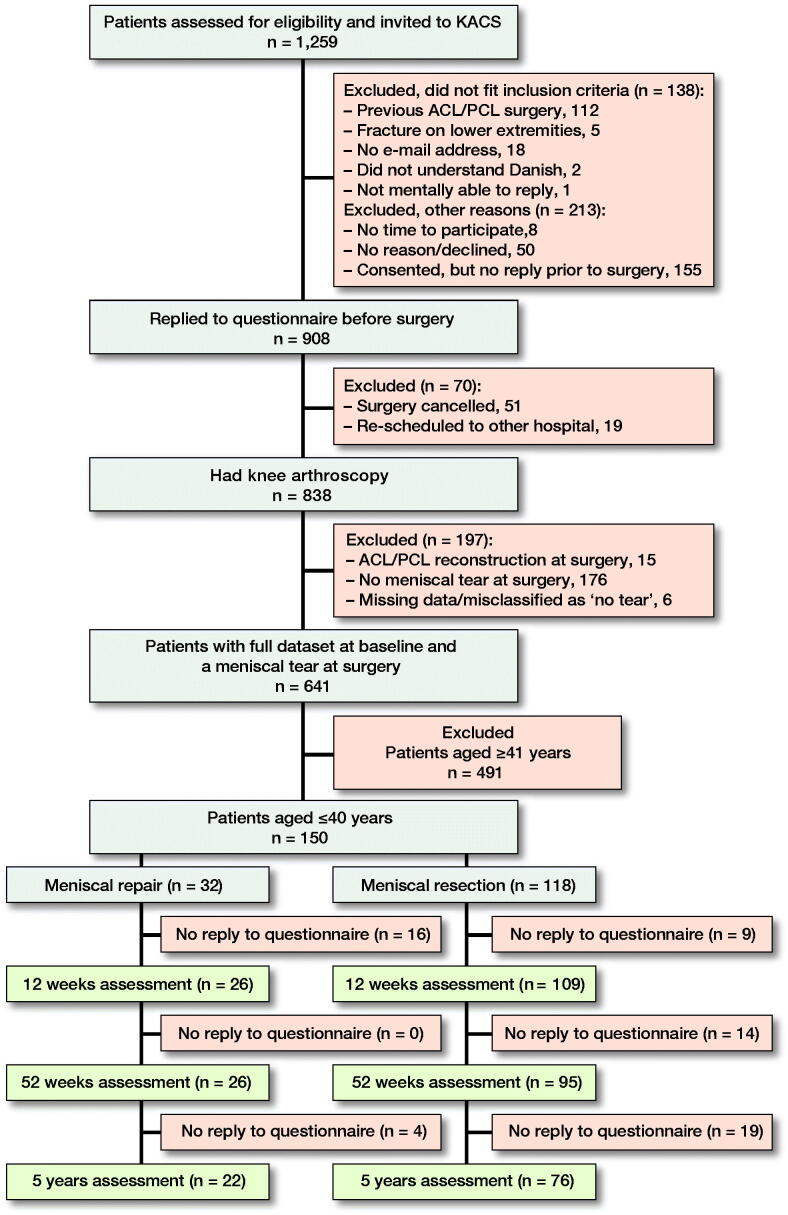
Flowchart of inclusion.

150 KACS patients (repair: n = 32 and APM: n = 118) aged 40 years or younger were included in this study (Figure 1). At the 52 weeks assessment, 29 (19%) patients were lost to follow-up (nRepair = 6 and nAPM = 23). Those lost to follow up among patients who had APM were slightly younger and had worse KOOS scores, whereas the KOOS scores among patients who had repair did not differ from those assessed at follow-up (Supplementary Table A1). Patients who had repair were marginally younger than the APM group, had less cartilage damage (Table 1), and differed in most meniscal pathologies (Table 2), whereas KOOS scores were comparable between the two groups.

**Table 1.** Baseline patient characteristics. Values are count (%) unless otherwise specified

	Repair	APM	Compara-
Factor	(n = 32)	(n = 118)	bility: SMD **^a^**
Age, mean (SD) [range]	26 (6) [18–38]	32 (7) [18–40]	0.76
Female	10 (31)	40 (34)	0.05
BMI, mean (SD) [range]	26 (3.2) [21–33]	27 (4.4) [19–44]	0.17
Participation in physical activity prior to injury		0.34	
Sport at competitive level	11 (34)	24 (20)	
Recreational sport	9 (28)	36 (31)	
Light sport	5 (16)	18 (15)	
Heavy household work	3 (9)	15 (13)	
Light household work	4 (13)	20 (17)	
Minimal household work	0 (0)	4 (3)	
No household work	0 (0)	1 (1)	
Symptom onset **^b^**			0.12
Slowly evolved over time	6 (19)	23 (19)	
Semi-traumatic	9 (28)	42 (36)	
Traumatic	17 (53)	53 (45)	
Duration of symptoms			0.18
0–3 months	9 (28)	32 (27)	
4–6 months	8 (25)	16 (14)	
7–12 months	6 (19)	25 (21)	
13–24 months	3 (9)	17 (14)	
	6 (19)	28 (24)	
KOOS scores, mean (SD) [range]			
KOOS_4_	50 (18) [13–83]	47 (16) [3–87]	0.12
Pain	62 (21) [8–100]	58 (20) [0–97]	
Symptoms	60 (20) [21–93]	61 (19) [11–100]	
ADL	73 (17) [34–99]	69 (20) [7–100]	
Sport/Rec	34 (27) [0–90]	30 (22) [0–90]	
QoL	45 (17) [0–75]	39 (16) [0–75]	

APM: arthroscopic partial meniscectomy, SD: standard deviation, BMI: body mass index, KOOS: Knee injury and Osteoarthritis Outcome Score, ADL: activities of daily living, Sport/rec: sport and recreational activities, QoL: knee-related quality of life.

**^a^**SMD = Standardized mean difference. Comparability is measured in SD units (derived from Kruskal–Wallis 2-sample test). An SMD of 0.5 or higher indicates the variable may be a confounding factor.

**^b^** Symptom onset defined by patient as: “The pain/problems have slowly developed over time” or “As a result of a specific incident (i.e., kneeling, sliding, and/or twisting of the knee or the like” (i.e., semi-traumatic) or “As a result of a violent incident (i.e., during sports, a crash, or collision or the like)” (i.e., traumatic).

**Table 2.** Surgical procedures and findings. Values are count (%) unless otherwise specified

	Repair	APM	Compara-
Factor	(n = 32)	(n = 118)	bility: SMD **^a^**
Type of repair surgery:			
Rasping	1 (3)	–	
Suture	7 (22)	–	
Arrow	1 (3)	–	
Anchor + suture	5 (16)	–	
More than one	18 (56)	–	
Type of repair technique **^b^**			
All-inside	17 (53)	–	
Inside-out	1 (3)	–	
Outside-in	1 (3)	–	
Amount resected (%),			
median (IQR) **^c^**	5 (5–10)	20 (10–29)	
Compartment			
Medial	23 (72)	69 (58)	0.23
Lateral	3 (9)	44 (37)	0.49
Both	6 (19)	5 (4)	0.25
Tear depth **^d^**			0.06
Partial	10 (31)	40 (34)	
Complete	22 (69)	75 (64)	
Tear type			
Longitudinal-vertical	28 (88)	33 (28)	0.80
Horizontal	1 (3)	5 (4)	0.02
Radial	0 (0)	4 (3)	0.06
Vertical flap	0 (0)	26 (22)	0.38
Horizontal flap	0 (0)	10 (8)	0.15
Complex	0 (0)	21 (18)	0.31
More than one tear type	3 (9)	19 (16)	0.12
Circumferential location **^d,e^**			0.81
Zone 1	25 (78)	30 (25)	
Zone 2	5 (16)	69 (58)	
Zone 3	1 (3)	16 (14)	
Radial location **^f^**			
Posterior	21 (66)	60 (51)	0.33
Posterior + mid-body	4 (13)	24 (20)	0.03
Mid body	3 (9)	14 (12)	0.04
Anterior + mid-body	0 (0)	6 (5)	0.12
Anterior	1 (3)	7 (6)	0.09
All	1 (3)	7 (6)	0.04
Meniscal tissue quality			0.50
Non-degenerative	32 (100)	84 (71)	
Degenerative	0 (0)	28 (24)	
Undetermined	0 (0)	6 (5)	
ACL status **^e^**			0.42
Intact	19 (61)	99 (84)	
Partial rupture **^g^**	2 (6)	7 (6)	
Total rupture **^g^**	10 (32)	12 (10)	
ICRS cartilage grade			
Medial compartment ≥ 2 **^h^**	0 (0)	17 (14)	0.30
Lateral compartment ≥ 2 **^f^**	0 (0)	12 (10)	0.45
Patellofemoral			
compartment ≥ 2 **^f,h^**	1 (3)	11 (11)	0.34

APM: arthroscopic partial meniscectomy,

ACL: Anterior cruciate ligament,

ICRS: International Cartilage Repair Society grading system.

**^a^**See Table 1.

**^b^**Missing data for 13 observations in repair group.

**^c^**Missing data for 5 observations in APM group. Data for repair group is only for the 8 patients who also had APM.

**^d^**Missing data for 3 observations in APM group.

**^e^**Missing data for 1 observation in repair group.

**^f^**Missing data for 2 observations in repair group.

**^g^**Non-reconstructed.

**^h^**Missing data for 2 observations in APM group.

**Figure 2.**
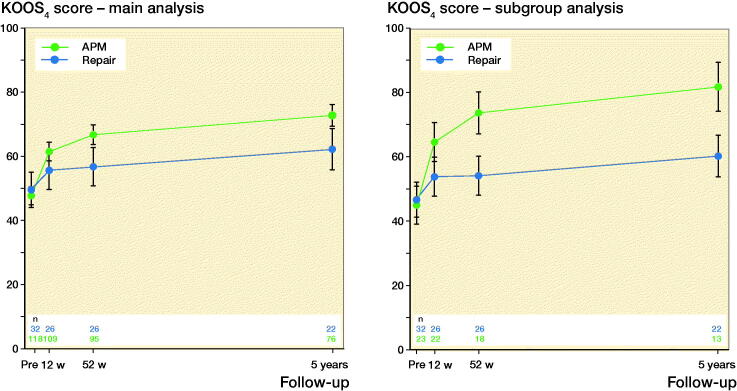
Least squares means for the Knee injury and Osteoarthritis Outcome Scores (KOOS)_4_ assessed before arthroscopic meniscal surgery, and at 12 weeks, 52 weeks, and median 5 years’ follow-up for patients having had meniscal repair or arthroscopic partial meniscectomy (APM). Data from main analysis and subgroup analysis (harmonized tear types) were adjusted for age, sex, body mass index (BMI), and preoperative KOOS_4_ score. Bars indicate 95% confidence intervals.

In the main analysis, both the repair and APM group improved in KOOS_4_ scores from before to 52 weeks after surgery (least square means 7 and 19, respectively; adjusted mean difference –12 [CI –19 to –4]) (Table 3). Both groups improved further from 52 weeks to 5 years after surgery with the difference in KOOS_4_ scores between the 2 groups being constant (Figure 2 and Supplementary Table A2). Similar findings were observed for all KOOS subscales (Table 3). All sensitivity analyses essentially yielded similar results to the main analyses (Supplementary Tables A3 to A6), but when excluding those who had had subsequent surgery on the index knee, the difference in change between groups varied considerably from before to 52 weeks after surgery (adjusted mean difference –22 [CI –34 to –9]), which was reduced at 5 years (adjusted mean difference –9 [CI –21 to 3]) (Supplementary Table A7).

**Table 3.** Change (95% confidence interval) in Knee injury and Osteoarthritis Outcome Scores (KOOS) from baseline prior to surgery to 12 weeks, 52 weeks, and median 5 years’ follow–up for patients who had had meniscal repair or APM performed

Factor	Repair	APM	Difference
**KOOS scores, unadjusted**			
Change from baseline at 12 weeks			
n	26	109	
KOOS_4_	5.8 (–1.0 to 13)	14 (10 to 17)	–7.9 (–16 to –0.3)
Pain	6.9 (0.1 to 14)	15 (11 to 18)	–7.9 (–16 to –0.3)
Symptoms	5.4 (–1.7 to 13)	11 (7.0 to 14)	–5.2 (–13 to 2.8)
ADL	7.0 (1.1 to 13)	13 (9.5 to 15)	–5.4 (–12 to 1.2)
Sport/Rec	8.0 (–2.0 to 18)	18 (13 to 23)	–10 (–21 to 1.2)
QoL	3.0 (–5.0 to 11)	12 (7.5 to 16)	–8.5 (–18 to 0.4)
Change from baseline at 52 weeks			
n	26	95	
KOOS_4_	7.1 (0.3 to 14)	20 (16 to 23)	–12 (–20 to –4.7)
Pain	7.5 (0.6 to 14)	18 (14 to 22)	–11 (–18 to –2.7)
Symptoms	3.5 (–3.7 (11)	15 (11 to 19)	–12 (–20 to –3.5)
ADL	6.6 (0.7 to 13)	15 (12 to 18)	–8.3 (–15 to –1.6)
Sport/Rec	14 (4.0 to 24)	27 (21 to 32)	–13 (–24 to –1.2)
QoL	3.6 (–4.4 to 12)	18 (14 to 23)	–15 (–24 to –5.7)
Change from baseline at 5 years			
n	22	76	
KOOS_4_	13 (5.3 to 20)	25 (21 to 29)	–13 (–21 to –4.5)
Pain	13 (6.0 to 21)	23 (20 to 27)	–10 (–18 to –1.8)
Symptoms	10 (2.4 to 18)	19 (15 to 23)	–8.7 (–17 to –0.2)
ADL	12 (5.3 to 18)	19 (15 to 22)	–6.9 (–14 to 0.3)
Sport/Rec	19 (7.9 to 29)	33 (27 to 38)	–14 (–26 to –2.1)
QoL	8.6 (0.1 to 17)	26 (22 to 31)	–18 (–27 to –7.9)
**KOOS scores, adjusted ^a^**			
Change from baseline at 12 weeks			
n	26	109	
KOOS_4_	6.1 (–0.6 to 13)	14 (10 to 17)	–7.7 (–15 to –0.2)
Pain	7.1 (0.4 to 14)	15 (12 to 18)	–7.7 (–15 to –0.2)
Symptoms	5.6 (–1.4 to 13)	11 (7.1 to 14)	–5.0 (–13 to 2.8)
ADL	7.1 (1.3 to 13)	12 (9.5 to 15)	–5.3 (–12 to 1.2)
Sport/Rec	8.2 (–1.7 to 18)	18 (13 to 23)	–9.8 (–21 to 1.3)
QoL	3.3 (–4.6 to 11)	12 (7.7 to 16)	–8.4 (–17 to 0.4)
Change from baseline at 52 weeks			
n	26	95	
KOOS_4_	7.2 (–0.5 to 14)	19 (16 to 23)	–12 (–19 to –4.3)
Pain	7.4 (0.7 to 14)	18 (14 to 21)	–10 (–18 to –2.6)
Symptoms	3.6 (–3.4 to 11)	15 (11 to 18)	–11 (–19 to –3.0)
ADL	6.7 (0.9 to 13)	15 (12 to 18)	–8.0 (–15 to –1.4)
Sport/Rec	15 (4.6 to 24)	26 (21 to 31)	–12 (–23 to –0.5)
QoL	3.5 (–4.4 to 11)	18 (14 to 22)	–15 (–24 to 5.9)
Change from baseline at 5 years			
n	22	76	
KOOS_4_	13 (5.6 to 20)	25 (21 to 29)	–12 (–20 to –4.4)
Pain	14 (6.4 to 21)	23 (20 to 27)	–9.8 (–18 to –1.7)
Symptoms	10 (2.9 to 18)	19 (15 to 23)	–8.2 (–17 to 0.1)
ADL	12 (5.9 to 18)	19 (15 to 22)	–6.6 (–14 to 0.4)
Sport/Rec	19 (8.1 to 29)	33 (27 to 38)	–14 (–26 to –2.3)
QoL	8.7 (0.4 to 17)	26 (22 to 30)	–17 (–27 to –7.7)

**^a^** Adjusted for age, sex, BMI, and preoperative KOOS score.

For abbreviations, see Table 1.

In the subgroup analysis aiming to compare patients with similar meniscal pathology in the 2 groups, the difference in improvement between the 2 groups from before to 52 weeks after surgery were larger than in the main analysis, in favor of the APM group (adjusted mean difference –21 [CI –31 to –11]), which was sustained at 5 years (Figure 2 and ­Supplementary Table A8 and A9). As in the main analysis, the difference when excluding those having had subsequent surgery was most pronounced at 52 weeks, while the repair group improved more from 52 weeks to 5 years than the APM group (Supplementary Table A10).

Patients who had repair were more likely to report having had knee problems and subsequent surgery at 52 weeks and 5 years after surgery. For satisfaction (PASS) and treatment failure, wide confidence intervals precluded interpretation of possible difference in proportions between the 2 groups (Table 4).

**Table 4.** Proportion of patients reporting acceptable symptoms state and treatment failure among those with unsatisfactory symptom state, and patients reporting having had subsequent knee surgery among those reporting knee problems after surgery

	At 52 weeks after initial surgery				At median 5 years after initial surgery			
	Repair (n = 26) yes/no	APM (n = 95) yes/no	Relative risk (95% CI)	Risk difference (95% CI)	Repair (n = 22) yes/no	APM (n = 75 **^a^**) yes/no	Relative risk (95% CI)	Risk difference (95% CI)
Satisfied with current knee function (PASS)	10/16	56/39	0.65 (0.39–1.1)	–0.20 (–0.42 to 0.01)	12/10	50/25	0.82 (0.54–1.2)	–0.12 (–0.36 to 0.11)
Treatment failure	6/10	17/22	0.86 (0.41–1.8)	–0.06 (–0.34 to 0.22)	5/5	5/20	2.5 (0.92–6.8)	0.30 (–0.05 to 0.65)
Knee problems								
after surgery	26/0	74/21	1.3 (1.2–1.4)	0.22 (0.14 to 0.30)	22/0	66/9	1.1 (1.1–1.2)	0.12 (0.05 to 0.19)
Re–surgery	6/20	9/65	1.9 (0.75–4.8)	0.11 (–0.07 to 0.29)	12/10	14/52	2.6 (1.4–4.7)	0.33 (0.10 to 0.56)

APM: arthroscopic partial meniscectomy, PASS: patient acceptable symptom state. CI: confidence interval.

**^a^** Data missing for 1 observation.

## Discussion

We found that patients undergoing repair improved less in patient-reported outcomes from before to around 5 years after surgery than patients having APM. The difference was mainly driven by larger improvements within the first year after surgery, while the groups improved equally in the period from 1 to approximately 5 years post-surgery. These results were consistent in all subgroup and sensitivity analyses. More patients in the repair group reported knee problems after the initial surgery and subsequent surgery to the index knee at 1- and 5-years’ follow-up compared with the APM group, although the difference in subsequent surgery at 1 year was not statistically significant.

To our knowledge, this is the first prospective study with pre-specified outcomes investigating changes in patient-reported outcomes after meniscal repair compared with APM, providing the most solid data so far in the absence of randomized trials. Previous attempts to compare meniscal repair and APM in patients with an isolated meniscal tear have been limited to small retrospective observational studies (Stein et al. 2010, Paxton et al. 2011, Lutz et al. 2015). They found no difference in absolute scores of self-reported symptoms or function at 2–5 years after surgery between the 2 procedures (Stein et al. 2010), but the repair group were found to have better scores at 6–13 years’ follow-up (Stein et al. 2010, Lutz et al. 2015). These results contrast with the present study, where the APM group at all follow-ups had better patient-reported outcomes than the repair group (Figure 2). Likely, more patients in the APM groups in previous studies that included older patients had clinical knee OA at follow-up compared with our study on young adults (Lutz et al. 2015). Any differences in patient-reported outcomes post-surgery between groups in previous retrospective studies might already have been present pre-surgery (Stein et al. 2010, Lutz et al. 2015).

The repair and APM groups had similar baseline KOOS_4_ scores, while the APM group had higher scores at all follow-ups than the repair group due to about a 12 points larger improvement from pre-surgery to 52 weeks and 5 years after surgery. A difference of this size is typically considered clinically relevant (Devji et al. 2017). Notably, none of the groups had reached population-based KOOS scores, especially in the subscales Sport/Rec and QOL (Paradowski et al. 2006).

Meniscal repair is a more complex procedure than APM and often requires an extended rehabilitation period. Previous studies have reported a reoperation rate for repair patients between 17% and 30% depending on time of follow-up, compared with a rate between 1% and 5% for APM patients (Paxton et al. 2011). Our findings are consistent with this, although the proportions who had subsequent surgery were larger at 5 years than previously reported. In the present study, the specific type of subsequent surgery to the index knee was not specified, which may mean that some of the subsequent surgery may not be related to the meniscus. In the sensitivity analyses excluding patients who had had subsequent surgery the difference observed in all analyses in improvement from before to 1 year after surgery between the repair and APM groups diminished from 1 to 5 years as a consequence of a larger improvement in the repair group. This supports the notion that the poorer outcomes from repair compared with APM might be due to the larger proportion having complications and subsequent surgery.

While APM may have better outcomes and fewer complications short-term, the procedure likely increases structural joint deterioration and risk of subsequent joint replacement (Collins et al. 2020). Therefore, meniscal repair is typically preferred when viable despite the risk of poorer short-term outcomes and complications (Kopf et al. 2020). The biomechanical advantages of procedures that preserve intra-articular contact area and stress are described (Baratz et al. 1986), but these theoretical benefits regarding the risk of OA have not yet been confirmed by clinical trial data. The limited evidence from observational studies supports the hypothesized benefits but suffers the same limitations as the present study, mainly confounding-by-indication. A recent Swedish registry study reported the incidence of OA after meniscal repair to be substantially elevated compared with the general population (Persson et al. 2018).

### Limitations

We are unable to draw conclusions regarding causality between the surgical procedures and the degree of improvement as this was an observational study. Like all previous studies the surgical procedure was not randomized but determined by the pathology (i.e., tear type), leading to selection bias. Although our findings were consistent and robust even after repeated adjustments (attempting to deal with prognostic imbalance to reduce the risk of bias in this observational setting), none of these adjustments can replace the lack of systematic bias in the distribution of both known and unknown prognostic factors offered by randomization.

At 52 weeks, loss to follow-up among the repair and APM groups was 19%. Those lost to follow-up in the APM group self-reported poorer KOOS scores before surgery compared with patients who remained in the study. However, the use of mixed models that include all patients with and without missing data at any time point should give unbiased results under the assumption of missing at random. To explore the robustness of deviations from the missing at random assumption, we assessed the impact of missing data in sensitivity analyses using different single-imputation techniques such as non-responder imputation (i.e., baseline observation carried forward), a best- and worst-case scenario, which yielded similar results for the main outcome (Supplementary Table A6).

In the study protocol the intent was to conduct this study on patients with traumatic tears only. However, as no clear consensus exists on the definition of traumatic and degenerative tears, we changed this to including all patients aged 18–40 years. A sensitivity analysis was performed using the planned definition of traumatic tears, but this did not change the interpretation of the results (Supplementary Table A11).

Although the use of repair surgery and technique varied considerably and possibly has affected the outcomes in the repair group, it is unlikely that it has had a substantial impact, as previous studies have reported comparable results between the different repair methods (Nepple et al. 2012).

We believe the results are generalizable to the majority of patients undergoing arthroscopic meniscal surgery as demographics of the included patients with regard to age and sex are similar to what has been reported for patients having meniscal surgery in Denmark and the United States (Montgomery et al. 2013, Thorlund et al. 2014). However, the results are not generalizable to patients with ACL/PCL reconstruction and a meniscal tear as these patients were excluded from this study. The proportion of individuals 40 years old or younger in the KACS cohort is a little lower than the corresponding number in all patients having had meniscal surgery in Denmark (Thorlund et al. 2014) and also only a small proportion in the present study were active at competitive level, indicating that we might have missed some young elite athletes.

## Conclusion

Patients who had had meniscal repair or APM improved in patient-reported outcomes after surgery; however, the repair group experienced clinically important smaller improvements at 1 year and 5 years post-surgery than patients who had had APM. The results highlight the need for randomized controlled trials comparing the short- and long-term outcomes of meniscal repair and APM on patient-reported outcomes and knee OA development.

## Supplementary Material

Supplemental Material

## Supplementary data

Tables A1–A11 are available as supplementary data in the online version of this article, http://dx.doi.org/10.1080/17453674.2021.1917826
