# Supplementary material for: Less improvement following meniscal repair compared with arthroscopic partial meniscectomy: a prospective cohort study of patient-reported outcomes in 150 young adults at 1- and 5-years’ follow-up
Source: Acta Orthop. 2021 Apr 30;92(5):589–96. doi: 10.1080/17453674.2021.1917826 (PMC8519526; doi:10.1080/17453674.2021.1917826)
Supplement: Supplemental Material [file IORT_A_1917826_SM2356.pdf]

Supplementary data

Supplementary Table A1. Baseline characteristics of patients who had meniscal repair or arthroscopic partial meniscectomy (APM) assessed at 52 weeks' follow-up and patients lost to follow-up at 52 weeks. Values are count unless otherwise specified

Factor	Repair Assessed at 52 weeks (n = 26)	Repair Lost to follow-up (n = 6)	APM Assessed at 52 weeks (n = 95)	APM Lost to follow-up (n = 23)
Age, mean (SD)	26 (6.3)	26 (4.6)	32 (7.1)	30 (7.0)
Female	9	1	29	11
BMI (SD)	26 (3.2)	26 (3.4)	26 (4.3)	28 (5.1)
Participation in physical activity prior to injury				
Sport at competitive level	6	5	21	3
Recreational sport	8	1	29	7
Light sport	5	0	12	6
Heavy household work	3	0	11	4
Light household work	4	0	18	2
Minimal household work	0	0	3	1
No household work	0	0	1	0
Symptom onset				
Slowly evolved over time	5	1	17	6
Semi-traumatic	8	1	36	6
Traumatic	13	4	42	11
Duration of symptoms				
0–3 months	7	2	25	7
4–6 months	7	1	14	2
7–12 months	6	0	20	5
13–24 months	2	1	14	3
> 24 months	4	2	22	6
KOOS scores, mean (SD)				
$KOOS_4$	50 (16)	49 (27)	49 (17)	39 (14)
Pain	63 (19)	57 (29)	61 (20)	48 (20)
Symptoms	60 (18)	60 (28)	63 (19)	50 (18)
ADL	74 (16)	70 (24)	71 (20)	61 (20)
Sport/Rec	33 (25)	38 (38)	32 (22)	23 (20)
QoL	45 (14)	41 (27)	40 (15)	37 (19)
Type of repair surgery				
Rasping	1	0	–	–
Suture	5	2	–	–
Arrow	1	0	–	–
Anchor + suture	5	0	–	–
More than one	14	4	–	–
Type of repair technique [a]				
All-inside	15	2	–	–
Inside-out	0	1	–	–
Outside-in	0	1	–	–
Amount resected [b]				
Compartment				
Medial	20	3	56	13
Lateral	1	2	35	9
Both	5	1	4	1
Tear depth [c]				
Partial	10	0	32	8
Complete	16	6	62	13
Tear type				
Longitudinal-vertical	22	6	25	8
Horizontal	1	0	4	1
Radial	0	0	2	2
Vertical flap	0	0	22	4
Horizontal flap	0	0	10	0
Complex	0	0	17	4
More than one tear type	3	0	15	4
Circumferential location [c,d]				
Zone 1	20	5	24	6
Zone 2	4	1	58	11
Zone 3	1	0	12	4
Radial location [e]				
Posterior	19	2	48	12
Posterior + mid-body	2	2	23	1
Mid body	2	1	9	5
Anterior + mid-body	0	0	5	1
Anterior	0	1	5	2
All	1	0	5	2
Meniscal tissue quality				
Non-degenerative	26	6	65	19
Degenerative	0	0	24	4
Undetermined	0	0	6	0
ACL status [d]				
Intact	16	3	82	17
Partial rupture [f]	2	0	5	2
Total rupture [f]	7	3	8	4
ICRS cartilage grade, n (%):				
Medial compartment ≥ 2 [g]	0	0	10	5
Lateral compartment ≥ 2 [d]	0	0	10	2
Patellofemoral compartment ≥ 2 [d,g]	0	0	9	4

APM: arthroscopic partial meniscectomy, SD: standard deviation, BMI: body mass index, KOOS: Knee injury and Osteoarthritis Outcome Score, ADL: activities of daily living, Sport/rec: sport and recreational activities, QoL: knee-related quality of life.
[a] Missing data for 13 observations in repair group.
[b] Missing data for 5 observations in APM group. Data for repair group are only for the 8 patients who also had APM.
[c] Missing data for 3 observations in APM group.
[d] Missing data for 1 observation in repair group.
[e] Missing data for 2 observations in repair group.
[f] Non-reconstructed
[g] Missing data for 2 observations in APM group.

Supplementary Table A2. Primary analysis: Knee injury and Osteoarthritis Outcome absolute scores (KOOS) at pre-surgery, 12 weeks, 52 weeks, and median 5 years' follow-up for patients who had had meniscal repair or arthroscopic partial meniscectomy (APM) performed. Values are mean (95% confidence interval)

Factor	Pre-surgery Repair (n = 32)	Pre-surgery APM (n = 118)	12 weeks Repair (n = 26)	12 weeks APM (n = 109)	52 weeks Repair (n = 26)	52 weeks APM (n = 95)	5 years Repair (n = 22)	5 years APM (n = 76)
KOOS scores, unadjusted								
$KOOS_4$	50 (43–57)	47 (44–51)	56 (49–63)	61 (57–64)	57 (50–65)	67 (63–70)	63 (55–70)	72 (68–76)
Pain	62 (55–69)	58 (55–62)	69(61–76)	73 (69–77)	69 (62–77)	76 (72–80)	75.(67–83)	82 (77–86)
Symptoms	60 (53–67)	60 (57–64)	65 (58–73)	71 (68–75)	64 (56–71)	76 (72–80)	70 (62–78)	79 (75–84)
ADL	73 (67–79)	69 (66–72)	80.(74–87)	81 (78–85)	80 (73–86)	84 (80–87)	85 (78–92)	87 (84–91)
Sport/Rec	34 (25–44)	30 (25–35)	42.(32–52)	48 (43–53)	48 (38–58)	57 (52–62)	53 (42–63)	63 (57–69)
QoL	45 (37–52)	39 (35–43)	48 (40–55)	51 (47–54)	48 (40–56)	57 (53–62)	53 (45–62)	65 (61–70)
KOOS scores, adjusted [a]								
$KOOS_4$	50 (44–55)	48 (45–51)	56 (50–62)	62 (59–64)	57 (51–63)	67 (64–70)	62 (56–69)	73 (69–76)
Pain	61 (56–67)	59 (56–62)	69 (63–74)	74 (71–77)	69 (63–75)	77 (74–80)	75 (69–81)	82 (79–86)
Symptoms	62 (56–67)	61 (58–64)	67 (61–73)	72 (69–74)	65 (59–71)	75 (72–78)	72 (66–79)	79 (76–83)
ADL	72 (67–77)	70 (67–72)	79 (74–84)	82 (80–85)	79 (74–84)	84 (82–87)	84 (79–89)	88 (86–91)
Sport/Rec	33 (25–41)	31 (27–35)	41 (32–50)	49 (45–53)	47 (38–56)	57 (53–62)	51 (42–61)	64 (59–69)
QoL	42 (36–49)	40 (37–43)	46 (39–53)	52 (48–55)	46 (39–53)	58 (55–62)	51 (44–59)	66 (62–70)

[a] Adjusted for age, sex, BMI, and preoperative KOOS score.
For abbreviations, see Supplementary Table 1.

Supplementary Table A3. Sensitivity analysis: effect of excluding patients who had both repair and arthroscopic partial meniscectomy (APM) performed on change in Knee injury and Osteoarthritis Outcome Scores ($KOOS_4$) from baseline prior to surgery to 12 weeks, 52 weeks, and median 5 years' follow-up for patients who had meniscal repair or APM performed. Values are mean (95% confidence interval)

Change from baseline	$KOOS_4$ scores, unadjusted Repair	APM	Difference	$KOOS_4$ scores, adjusted [a] Repair	APM	Difference
at 12 weeks (n = 19/109)	6.1 (–1.8 to 14)	14 (10 to 17)	–7.6 (–16 to 1.0)	6.1 (–1.6 to 14)	14 (10 to 17)	–7.6 (–16 to –0.8)
at 52 weeks (n = 19/95)	6.0 (–1.9 to 14)	19 (16 to 23)	–13 (–22 to –4.8)	5.9 (–1.9 to 14)	19 (16 to 23)	–13 (–22 to –4.7)
at 5 years (n = 17/76)	13 (4.6 to 21)	25 (21 to 29)	–12 (–22 to –3.4)	13 (4.5 to 21)	25 (21 to 29)	–13 (–21 to –3.6)

[a] Adjusted for age, sex, BMI, and preoperative KOOS score.

Supplementary Table A4. Sensitivity analysis: effect of excluding patients who had partial or total ACL rupture on change in Knee injury and Osteoarthritis Outcome Scores ($KOOS_4$) from baseline prior to surgery to 12 weeks, 52 weeks, and median 5 years' follow-up for patients who had meniscal repair or APM performed. Values are mean (95% confidence interval)

Change from baseline	$KOOS_4$ scores, unadjusted Repair	APM	Difference	$KOOS_4$ scores, adjusted [a] Repair	APM	Difference
at 12 weeks (n = 14/92)	6.2 (–2.9 to 15)	15 (11 to 19)	–8.7 (–19 to 1.2)	5.9 (–3.1 to 15)	15 (11 to 19)	–9.2 (–19 to 0.5)
at 52 weeks (n = 16/82)	1.8 (–6.9 to 11)	20 (17 to 24)	–19 (–28 to –9.0)	1.7 (–6.9 to 10)	20 (16 to 24)	–18 (–28 to –8.9)
at 5 years (n = 14/65)	11 (1.9 to 20)	26 (22 to 30)	–15 (–25 to –4.7)	1.0 (2.0 to 20)	26 (22 to 30)	–15 (–25 to –4.8)

[a] Adjusted for age, sex, BMI, and preoperative KOOS score.

Supplementary Table A5. Sensitivity analysis: effect of additional adjustment for surgical findings with standardized mean differences (SMD) ≥ 0.50 [a] between surgery groups (tear pattern, tear location, and tissue quality) on change in Knee injury and Osteoarthritis Outcome Scores ($KOOS_4$) from baseline prior to surgery to 12 weeks, 52 weeks, and median 5 years' follow-up for patients who had meniscal repair or APM performed. Values are mean (95% confidence interval)

Change from baseline	$KOOS_4$ scores, adjusted [b] Repair	APM	Difference
at 12 weeks (n = 25/106)	6.7 (–0.1 to 14)	14 (11 to 18)	–7.5 (–15 to 0.7)
at 52 weeks (n = 25/94)	7.8 (1.0 to 15)	19 (16 to 23)	–12 (–19 to –3.9)
at 5 years (n = 21/74)	14 (6.4 to 21)	25 (22 to 29)	–12 (–20 to –3.5)

[a] Comparability is measured in SD units (derived from Kruskal–Wallis 2-sample test). An SMD ≥0.5 indicates the variable may be a confounding factor. (Imbens G W, Rubin D B. Causal inference for statistics, social, and biomedical sciences: an introduction. Cambridge: Cambridge University Press; 2015.)
[b] Adjusted for age, sex, BMI, preoperative KOOS score, meniscal tear pattern, tear location, and tissue quality.

Supplementary Table A6. Sensitivity analyses: effect of null-responder and best/worst case scenario imputation of missing data on change in Knee injury and Osteoarthritis Outcome Score ($KOOS_4$) from baseline prior to surgery to 12 weeks, 52 weeks, and median 5 years' follow-up for patients who had meniscal repair or arthroscopic partial meniscectomy (APM) performed. Values are mean (95% confidence interval)

Change from baseline	$KOOS_4$ scores, unadjusted Repair	APM	Difference	$KOOS_4$ scores, adjusted [a] Repair	APM	Difference
Sensitivity analysis: 1						
at 12 weeks (n = 32/118)	5.1 (–1.3 to 12)	13 (9.4 to 16)	–7.6 (–15 to –0.3)	5.1 (–1.3 to 12)	13 (9.4 to 16)	–7.6 (–15 to –0.3)
at 52 weeks (n = 32/118)	6.0 (–0.4 to 13)	15 (12 to 18)	–9.0 (–16 to –1.7)	6.0 (–0.4 to 13)	15 (12 to 18)	–9.0 (–16 to –1.7)
at 5 years (n = 32/118)	9.0 (2.5 to 16)	16 (13 to 20)	–7.3 (–15 to –0.0)	9.0 (2.5 to 16)	16 (13 to 20)	–7.3 (–15 to –0.5)
Sensitivity analysis: 2						
at 12 weeks (n = 32/118)	9.1 (2.6 to 16)	13 (9.2 to 16)	–3.5 (–11 to 3.9)	9.1 (2.6 to 16)	13 (9.2 to 16)	–3.5 (–11 to 3.9)
at 52 weeks (n = 32/118)	9.8 (3.3 to 16)	18 (14 to 21)	–7.7 (–15 to –0.3)	9.8 (3.3 to 16)	18 (14 to 21)	–7.7 (–15 to –0.3)
at 5 years (n = 32/118)	19 (12 to 26)	21 (18 to 24)	–2.0 (–9.4 to 5.4)	19 (12 to 26)	21 (18 to 24)	–2.0 (–9.4 to 5.4)
Sensitivity analysis: 3						
at 12 weeks (n = 32/118)	3.2 (–3.6 to 10)	15 (11 to 18)	–12 (–19 to –4.0)	3.2 (–3.6 to 10)	15 (11 to 18)	–12 (–19 to –4.0)
at 52 weeks (n = 32/118)	5.4 (–1.4 to 12)	24 (20 to 27)	–18 (–26 to –11)	5.4 (–1.4 to 12)	24 (20 to 27)	–18 (–26 to –11)
at 5 years (n = 32/118)	6.4 (–0.3 to 13)	32 (30 to 35)	–25 (–33 to –17)	6.4 (–0.3 to 13)	32 (30 to 35)	–25 (–33 to –17)

[a] Adjusted for age, sex, BMI, and preoperative KOOS score.
[b] 25th and 75th percentile values calculated from patients with available data at 12 weeks, 52 weeks, and ~5 years in the repair and APM group, respectively.
Mixed models that include all patients with and without missing data at any time point should give unbiased results under the assumption of missing at random. However, we conducted a number of sensitivity analysis to test the robustness of the results including null-responder and best/worst case scenario imputation of $KOOS_4$ scores. (Vittinghoff E. Regression methods in biostatistics: linear, logistic, survival, and repeated measures models. 2nd ed. New York: Springer; 2012, p 509). Sensitivity analyses:
1 Null-responder imputation (i.e., baseline observation carried forward).
2 Imputation of 75 percentile and 25 percentile $KOOS_4$ value for patients lost to follow-up at 12 weeks, 52 weeks, and ~5 years in repair group (i.e., assuming best case) and APM group (i.e., assuming worst case), respectively [b].
3 Imputation of 75 percentile and 25 percentile $KOOS_4$ value for patients lost to follow-up at 12 weeks, 52 weeks, and ~5 years in APM group (i.e., assuming best case) and repair group (i.e., assuming worst case), respectively [b].

 Acta Orthopaedica 2021; 92 (DOI: 10.1080/17453674.2021.1917826)

Supplementary Table A7. Sensitivity analysis: effect of excluding patients who had subsequent surgery on index knee during the 5-year follow-up on change in Knee injury and Osteoarthritis Outcome Scores ($KOOS_4$) from baseline prior to surgery to 12 weeks, 52 weeks, and median 5 years; follow-up for patients having had meniscal repair or APM performed. Values are mean (95% confidence interval)

Change	$KOOS_4$ scores, unadjusted			$KOOS_4$ scores, adjusted [a]		
from baseline	Repair	APM	Difference	Repair	APM	Difference
at 12 weeks (n = 16/96)	3.3 (–8.3 to 15)	15 (10 to 20)	–12 (–24 to 0.8)	3.0 (–8.5 to 15)	15 (10 to 20)	–12 (–24 to 0.5)
at 52 weeks (n = 14/82)	1.2 (–10 to 13)	24 (19 to 28)	–23 (–35 to –10)	1.6 (–9.9 to 13)	23 (19 to 28)	–22 (–34 to –9.3)
at 5 years (n = 10/62)	20 (8.3 to 31)	29 (24 to 33)	–9.0 (–21 to 3.1)	20 (8.3 to 31)	29 (24 to 33)	–9.0 (–21 to 3.1)

[a] Adjusted for age, sex, BMI, and preoperative KOOS score.

Supplementary Table A8. Subgroup analysis: change in KOOS from baseline prior to surgery to 12 weeks, 52 weeks, and median 5 years' follow-up for patients who had meniscal repair or arthroscopic partial meniscectomy (APM) performed excluding patients in the APM group who had a meniscal tear pattern typically considered ineligible for repair (see foot note). Values are mean (95% confidence interval)

Factor	Repair	APM	Difference
**KOOS scores, unadjusted**			
Change from baseline at 12 weeks			
n	26	22	
$KOOS_4$	5.8 (–0.7 to 12)	19 (12 to 27)	–14 (–23 to –3.8)
Pain	6.9 (0.3 to 14)	21 (13 to 28)	–14 (–24 to –4.9)
Symptoms	5.4 (–1.7 to 13)	10 (2.1 to 18)	–4.7 (–15 to 6.0)
ADL	7.1 (1.7 to 12)	17 (11 to 22)	–9.5 (–17 to –1.5)
Sport/Rec	8.0 (–2.0 to 18)	28 (17 to 39)	–20 (–35 to –4.7)
QoL	3.0 (–4.6 to 11)	19 (10 to 27)	–16 (–27 to –4.5)
Change from baseline at 52 weeks			
n	26	18	
$KOOS_4$	7.1 (0.6 to 14)	29 (22 to 37)	–22 (–32 to –12
Pain	7.5 (0.9 to 14)	29 (21 to 37)	–21 (–31 to –11
Symptoms	3.5 (–3.7 to 11)	23 (15 to 32)	–20 (–31 to –8.6)
ADL	6.6 (1.3 to 12)	22 (16 to 29)	–16 (–24 to –7.5)
Sport/Rec	14 (4.0 to 24)	39 (28 to 51)	–25 (–41 to –9.8)
QoL	3.6 (–4.0 to 11)	26 (17 to 35)	–23 (–35 to –11)
Change from baseline at 5 years			
n	22	13	
$KOOS_4$	13 (5.6 to 19)	37 (28 to 45)	–24 (–35 to –13)
Pain	13 (6.3 to 20)	34 (25 to 42)	–20 (–32 to –9.1)
Symptoms	10 (2.4 to 18)	25 (15 to 34)	–15 (–27 to –2.4)
ADL	12 (5.9 to 17)	25 (18 to 32)	–14 (–23 to –4.7)
Sport/Rec	19 (7.9 to 29)	51 (37 to 64)	–32 (–49 to –15)
QoL	8.6 (0.6 to 17)	39 (29 to 49)	–30 (–43 to –17)
**KOOS scores, adjusted** [a]			
Change from baseline at 12 weeks			
n	26	22	
$KOOS_4$	6.1 (–0.3 to 12)	20 (12 to 27)	–14 (–23 to –4.0
Pain	9.7 (2.6 to 17)	21 (14 to 28)	–11 (–21 to –0.9)
Symptoms	5.7 (–1.3 to 13)	10 (2.4 to 18)	–4.6 (–15 to 5.9)
ADL	7.1 (1.9 to 12)	17 (11 to 22)	–9.4 (–17 to –1.6)
Sport/Rec	8.1 (–1.7 to 18)	28 (17.to 39)	–20 (–35 to –5.3)
QoL	3.3 (–4.1 to 11)	19 (11 to 27)	–16 (–27 to –4.6)
Change from baseline at 52 weeks			
n	26	18	
$KOOS_4$	7.4 (1.0 to 14)	29 (21 to 36)	–21 (–31 to –11)
Pain	6.9 (–0.2 to 14)	28 (20 to 35)	–20 (–30 to –10)
Symptoms	3.8 (–3.2 to 10.8)	23 (15 to 31)	–19 (–30 to –8.2)
ADL	6.8 (1.5 to 12.0)	22 (15 to 28)	–15 (–23 to –6.7)
Sport/Rec	15 (4.9 to 24.6)	39 (27 to 51)	–24 (–39 to –8.6
QoL	26 (17 to 35)	–22 (–34 to –11)	9.2 (1.3 to 17)
Change from baseline at 5 years			
n	22	76	
$KOOS_4$	13 (6.3 to 20)	37 (28 to 45)	–24 (–35 to –13)
Pain	15 (7.4 to 22)	33 (25 to 42)	–20 (–31 to –8.6)
Symptoms	11 (3.4 to 18)	24 (15 to 34)	–14 (–25 to –1.6)
ADL	12 (6.7 to 18)	25 (18 to 32)	–13 (–22 to –4.1)
Sport/Rec	19 (8.7 to 30)	53 (40 to 66)	–34 (–50 to –17)
QoL	9.2 (1.3 to 17)	38 (28 to 48)	–29 (–42 to –17)

[a] Adjusted for age, sex, BMI, and preoperative KOOS score.
For abbreviations, see Supplementary Table 1.
To make the 2 groups as comparable as possible we conducted a subgroup analysis excluding patients in the APM group with tears that are typically considered ineligible for meniscal repair (i.e., tears not being non-degenerative longitudinal-vertical tears located in the red–red or red–white zone as recorded by the surgeon at arthroscopy using the ISAKOS questionnaire). (Burns et al. Meniscus repair and transplantation techniques. J Knee Surg 2011; 24(3): 167-74. Karia et al. Current concepts in the techniques, indications and outcomes of meniscal repairs. Eur J Orthop Surg Traumatol: 2019; 29 (3): 509-20.)

Supplementary Table A9. Subgroup analyses: Knee injury and Osteoarthritis Outcome absolute scores (KOOS) at pre-surgery, 12 weeks, 52 weeks, and median 5 years' follow-up for patients who had meniscal repair or arthroscopic partial meniscectomy (APM) performed excluding patients in the APM group who had a meniscal tear pattern typically considered ineligible for repair (i.e., not having a non-degenerative vertical-longitudinal tear located in the red–red or red–white zone of the meniscus). Values are mean (95% confidence interval)

	Pre-surgery		12 weeks		52 weeks		5 years	
Factor	Repair (n = 32)	APM (n = 23)	Repair (n = 26)	APM (n = 22)	Repair (n = 26)	APM (n = 18)	Repair (n = 22)	APM (n = 13)
KOOS scores, unadjusted								
$KOOS_4$	50 (43–57)	40 (32–48)	56 (9–63.0)	59 (51–67)	57 (50–64)	68 (60–78)	63 (55–70)	76 (67–86)
Pain	62 (55–69)	50 (42–58)	69 (61–76)	71 (62–79)	69 (62–77)	79 (70–88)	75 (67–83)	84 (74–93)
Symptoms	60 (53–67)	55 (47–64)	65 (58–73)	66 (57–74)	64 (56–71)	79 (70–88)	70 (62–78)	80 (70–90)
ADL	73 (67–79)	54 (57–71)	80 (74–86)	80 (73–88)	80 (74–86)	86 (79–94)	85(78–91)	89 (81–97)
Sport/Rec	34 (25–44)	22 (11–34)	42 (32–52)	50 (38–61)	48 (38–58)	62 (49–74)	53 (42–64)	73 (59–87)
QoL	45 (38–51)	31 (23–39)	48 (40–55)	50 (41–58)	48 (41–56)	57 (48–66)	53 (45–61)	70 (60–80)
KOOS scores, adjusted [a]								
$KOOS_4$	47 (42–52)	45 (39–51)	53 (48–59)	65 (59–71)	54 (49–60)	74 (67–80)	60 (54–66)	82 (74–89)
Pain	59 (54–64)	56 (50–61)	66 (61–72)	76 (70–82)	67 (61–72)	83 (77–90)	73 (67–79)	89 (81–96)
Symptoms	59 (54–64)	58 (52–64)	65 (59–70)	68 (62–75)	63 (57–69)	81 (74–88)	70 (64–76)	82 (74–91)
ADL	71 (67–75)	68 (63–73)	78 (74–83)	84 (79–89)	78 (74–82)	90 (84–95)	83 (79–88)	93 (87–100)
Sport/Rec	30 (23–38)	27 (19–36)	38 (30–47)	56 (47–65)	45 (37–53)	66 (56–76)	49 (41–58)	80 (68–91)
QoL	41 (35–46)	37 (30–45)	44 (38–50)	57 (49–64)	44 (38–51)	64 (56–71)	50 (43–57)	76 (67–85)

[a] Adjusted for age, sex, BMI, and preoperative KOOS score.
For abbreviations, see Supplementary Table 1.

Supplementary Table A10. Sensitivity analysis (in subgroup): effect of excluding patients who had subsequent surgery on index knee during the 5-year follow-up on change in Knee injury and Osteoarthritis Outcome Scores (KOOS) from baseline prior to surgery to 12 weeks, 52 weeks, and median 5 years' follow-up for patients who had meniscal repair or arthroscopic partial meniscectomy (APM) performed, excluding patients in the APM group who had a meniscal tear pattern typically considered ineligible for repair (i.e., not having a non-degenerative vertical-longitudinal tear located in the red–red or red–white zone of the meniscus). Values are mean (95% confidence interval)

Change	$KOOS_4$ scores, unadjusted			$KOOS_4$ scores, adjusted [a]		
from baseline	Repair	APM	Difference	Repair	APM	Difference
at 12 weeks (n = 9/10)	3.3 (–8.3 to 15)	20 (8.6 to 31)	–17 (–33 to –0.3)	3.2 (–8.4 to 15)	20 (8.6 to 31)	–17 (–33 to –0.5)
at 52 weeks (n = 9/8)	1.2 (–10 to 13)	33 (21 to 45)	–32 (–49 to –15)	1.8 (–9.8 to 13)	32 (20.to 44)	–30 (–47 to –14)
at 5 years (n = 10/10)	20 (8.3 to 31)	36 (24 to 47)	–16 (–32 to –0.2)	20 (8.3 to 31)	36 (24 to 47)	–16 (–32 to –0.2)

[a] Adjusted for age, sex, BMI, and preoperative KOOS score.

Supplementary Table A11. Sensitivity analysis investigating change in Knee injury and Osteoarthritis Outcome Scores ($KOOS_4$) from baseline prior to surgery to 12 weeks, 52 weeks and median 5 years follow-up for patients with a traumatic meniscal tear [a] having had meniscal repair or arthroscopic partial meniscectomy (APM) performed. Values are mean (95% confidence interval)

Change	$KOOS_4$ scores, unadjusted			$KOOS_4$ scores, adjusted [b]		
from baseline	Repair	APM	Difference	Repair	APM	Difference
at 12 weeks (n = 21/69)	3.4 (–4.4 to 11)	14 (9.6 to 18)	–11 (–20 to –1.6)	3.7 (–4.0 to 11)	14 (9.7 to 18)	–10 (–19 to –1.5)
at 52 weeks (n = 20/58)	6.8 (–1.2 to 15)	19 (14 to 23)	–12 (–21 to –2.5)	6.9 (–0.9 to 15)	18 (14 to 23)	–11 (–20 to –2.2)
at 5 years (n = 16/48)	12 (3.8 to 21)	24 (19 to 29)	–12 (–22 to –1.8)	13 (4.1 to 21)	24 (19 to 29)	–12 (–21 to –1.9)

[a] Traumatic meniscal tear defined as patients aged 18–34 replying that symptoms evolved as a result of a specific or violent incident, and patients aged 34–55 replying that symptoms evolved as a result of a violent incident. In the original study protocol (Thorlund et al. 2013) the intent was to conduct this study on patients with traumatic tears only. However, as no clear consensus exists on the definition of traumatic and degenerative tears, we changed this to including all patients aged 18–40 years. The results of the primary analysis of the study are shown using the originally planned inclusion criteria.
[b] Adjusted for age, sex, BMI, and preoperative KOOS score. Numbers are mean values (95% confidence interval).
